# Impact of high neutrophil‐to‐lymphocyte ratio on survival in hospitalized cancer patients with COVID‐19

**DOI:** 10.1002/cam4.5426

**Published:** 2022-11-13

**Authors:** Fernando A. Díaz‐Couselo, Santiago Flagel, Carla Nicolini, Sebastián Halac, Natalia Manzano, Marina Aguirre, Juan Rébora, Sandra Valle, Laura Noro, Chirayu Mohindroo, Florencia McAllister, Marcelo Zylberman

**Affiliations:** ^1^ Department of Internal Medicine Instituto Alexander Fleming Buenos Aires Argentina; ^2^ Department of Infectious Diseases Instituto Alexander Fleming Buenos Aires Argentina; ^3^ Microbiology Laboratory Instituto Alexander Fleming Buenos Aires Argentina; ^4^ Clinical Analysis and Molecular Medicine Laboratory Instituto Alexander Fleming Buenos Aires Argentina; ^5^ Department of Clinical Cancer Prevention The University of Texas MD Anderson Cancer Center Houston Texas USA; ^6^ Department of Internal Medicine Sinai Hospital of Baltimore Baltimore Maryland USA

**Keywords:** biomarkers, cancer, COVID‐19, inflammatory markers, neutrophil lymphocyte ratio, neutrophils, NLR

## Abstract

Neutrophil‐to‐lymphocyte ratio (NLR) has been studied as a prognostic factor for mortality in COVID‐19 patients. Our study aimed to evaluate the association between NLR at COVID‐19 diagnosis and survival during the following 90 days in hospitalized patients with solid cancer. Between May 2020 and June 2021, 120 patients were included in a retrospective cohort study. Univariable analysis showed patients with an NLR > 8.3 were associated with an increased risk of death (HR: 4.34; 95% CI: 1.74–10.84) compared to patients with NLR < 3.82 and with NLR ≥3.82 and ≤8.30 (HR: 2.89; 95% CI: 1.32–6.36). Furthermore, on multivariable analysis, NLR > 8.30 independently correlated with increased mortality. In patients with solid malignancies with COVID‐19, an NLR > 8.3 is associated with an increased risk of death.

## Graphical abstract

In patients with solid malignancies hospitalized with COVID‐19, an elevated NLR at hospital admission may be predictive of increased risk of death.

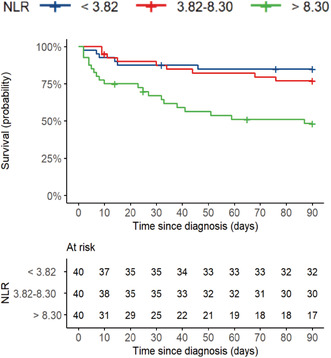

## 1 INTRODUCTION

COVID‐19 disease was declared a pandemic by the World Health Organization (WHO) in early 2020.^1^ In Wuhan, China, Yu et al. observed that the COVID‐19 infection rate among cancer patients was 0.79%, almost double than in the general population.^2^ In another study, which included 59,989 patients, Aschele et al., reported an infection rate of 0.68% in subjects with cancer.^3^ Patients with cancer had a higher risk of hospitalization, intensive care unit admission, and need for mechanical ventilation for COVID‐19 than the general population.^4^ A cohort study of 1590 COVID‐19 subjects, found that the requirement of ICU and mechanical ventilation was 3 to 5 times higher in cancer patients.^5^

Many studies have reported neutrophil‐to‐lymphocyte rate (NLR), as a biomarker for severity and mortality in COVID‐19 patients. Non‐survivors had a higher NLR on admission or diagnosis than survivors.^6^, ^7^, ^8^, ^9^, ^10^, ^11^ Cancer patients were underrepresented in majority of the studies, accounting for <4% of the study populations.^6^, ^8^, ^10^, ^12^, ^13^, ^14^

Risk factors associated with a poor prognosis in oncological patients include age,^15^, ^16^, ^17^ smoking history,^15^, ^18^, ^19^ comorbidities^15^ and gender.^15^, ^17^ Recent chemotherapy treatment was an inconsistent variable.^15^, ^18^, ^20^, ^21^ NLR, besides being cost effective and readily available, could also play a role in pathogenesis of COVID‐19.^22^ Barnes et al. reported results of the autopsy of 3 patients who died from COVID‐19 and showed the presence of abundant neutrophils, specifically in the lung tissue.^23^ Similar findings were also shown by other groups hence, highlighting the importance of neutrophils in severe COVID‐19 infection.^24^, ^25^

The aim of this study was to evaluate the association between NLR at COVID‐19 diagnosis and survival during the 90 days after diagnosis in a cohort of hospitalized patients with cancer and COVID‐19.

## 2 MATERIALS AND METHODS

We conducted a retrospective cohort study at the Instituto Alexander Fleming in Argentina in which we included patients with a history of solid neoplastic disease admitted to the hospital for COVID‐19, or who developed COVID‐19 during hospital admission for other causes, between May 2020 and June 2021. COVID‐19 was confirmed by reverse transcriptase polymerase chain reaction (rt‐PCR; Film Array and/or Gene Finder) or immunochromatography in all cases. Patients were excluded if COVID confirmation was done more than 7 days before admission. The study was approved by the Ethics Committee of the Instituto Alexander Fleming.

Demographic variables, comorbidities, underlying oncological disease, stage, and antineoplastic treatment during the 30 days prior to diagnosis were extracted from electronic medical records. Laboratory results within 48 h of COVID‐19 diagnosis were extracted from electronic laboratory reports. Follow‐up for survival time, within the 90 days after COVID‐19 diagnosis, was recorded. In case of censored data before day 90 since COVID‐19 diagnosis, researchers tried to contact the patient (or his/her family) for additional information. COVID‐19 was categorized as in‐hospital acquired if the diagnosis was made 48 h after admission. Details of statistical analysis and categorization of variables have been included in Supporting Information.

## 3 RESULTS

One hundred and twenty patients were included in the study. The most frequent tumors were breast (20.8%), lung (20%) and colorectal cancer (15.8%); (Table S1). Demographic details, Frequencies of comorbidities, chemotherapy exposure within 30 days of COVID‐19 diagnosis, in‐hospital acquired infection and laboratory results at diagnosis are summarized in Table S2. Six patients were lost during the follow‐up. Mortality rate was 29.2% (35 patients).

**FIGURE 1:**
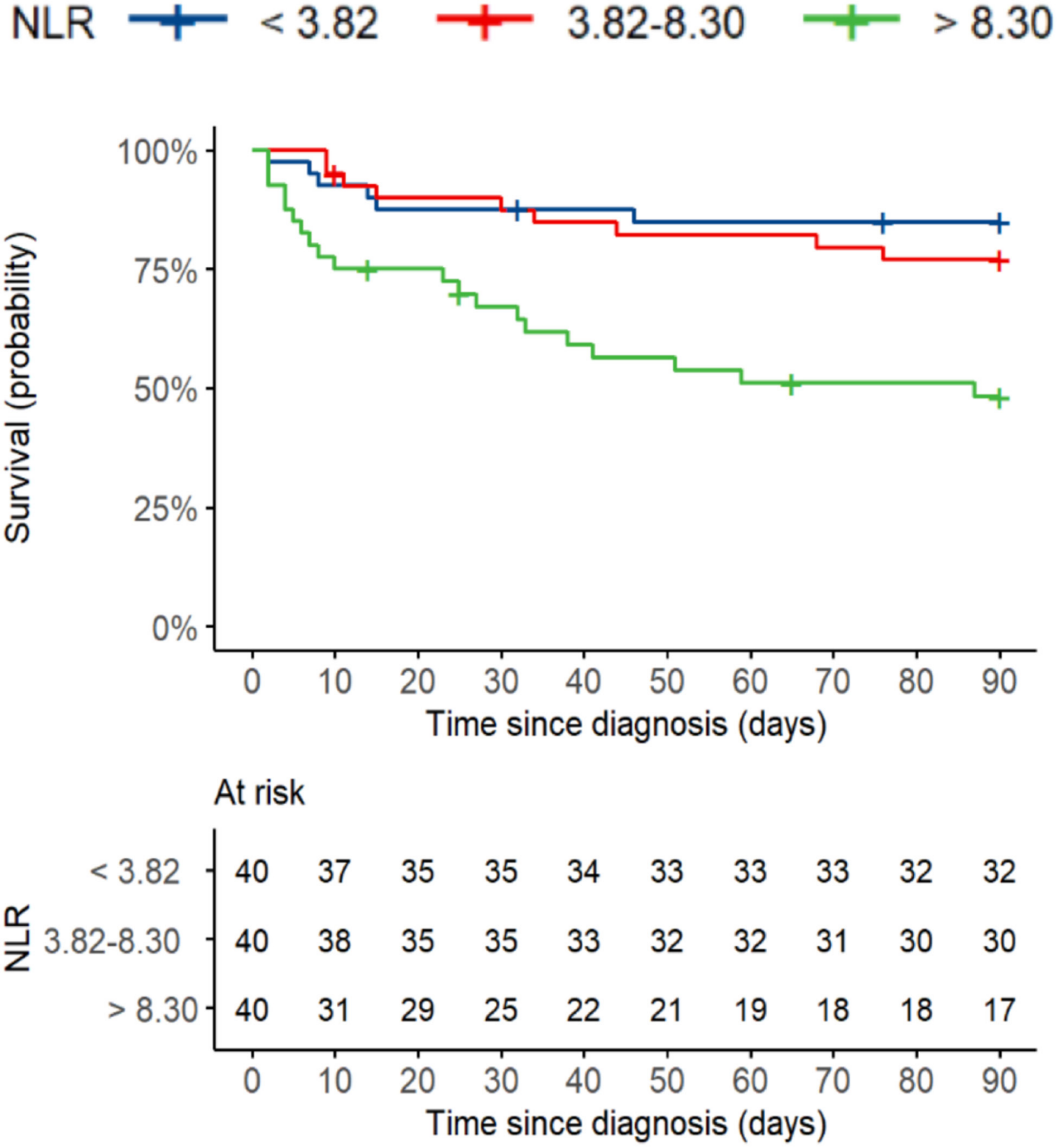
Kaplan–Meier plot of overall survival stratified by neutrophil‐to‐lymphocyte ratio (tertiles) following the 90 days of COVID‐19 diagnosis. NLR, neutrophil‐to‐lymphocyte ratio

Hazard ratios obtained from the univariable, and multivariable Cox models are summarized in Table 1 Patients with a NLR > 8.3 (third tertile) were associated with an increased risk of death (HR: 4.34; 95% CI: 1.74–10.84) compared with patients with NLR < 3.82 (first tertile) and patients with NLR between 3.82 and 8.3 (second tertile; HR: 2.89; 95% CI: 1.32–6.36). The Kaplan–Meier plot for the NLR tertiles is shown in Figure 1.

**TABLE 1:** Summary of univariable/multivariable analysis

Variable	Univariable		Multivariable	
	HR (95% CI)	*p*‐value	HR (95% CI)	*p*‐value
NLR^b^				
<3.82	Ref	—	Ref	—
3.82–8.30	1.50 (0.53–4.22)	0.4409	1.20 (0.41–3.49)	0.7378
>8.30	4.34 (1.74–10.84)	**0.0020**	3.33 (1.23–8.99)	**0.0178**
D‐dimer (ng/ml)				
<1105.30	Ref		Ref	—
1105.30–2411.87	3.59 (1.30–9.89)	**0.0134**	4.46 (1.49–13.39)	**0.0076**
>2411.87	3.79 (1.38–10.45)	**0.0099**	3.81 (1.25–11.64)	**0.0190**
Ferritin (ng/ml)				
<634.19	Ref		Ref	—
634.19–1499.03	1.21 (0.50–2.92)	0.672	1.27 (0.50–3.24)	0.6122
>1499.03	1.98 (0.87–4.52)	0.106	1.06 (0.42–2.68)	0.8999
LDH (U/L)				
>220	1.35 (0.63–2.88)	0.44	—	—
CRP (mg/L)				
<40.63	Ref		Ref	—
40.63–109.83	0.68 (0.26–1.78)	0.4293	0.47 (0.16–1.42)	0.1818
>109.83	1.92 (0.88–4.15)	0.0995	1.13 (0.44–2.86)	0.8004
Creatinine				
>UNL	2.22 (1.07–4.64)	**0.0331**	1.61 (0.62–4.19)	0.3333
Age ≥ 65 years	2.33 (1.19–4.59)	**0.0143**	1.68 (0.74–3.82)	0.2143
Male	1.54 (0.80–2.99)	0.2	—	—
Smoking history	1.68 (0.86–3.28)	0.13	1.27 (0.60–2.68)	0.5266
Diabetes	1.43 (0.62–3.27)	0.4	—	—
Hypertension	0.90 (0.45–1.82)	0.778	—	—
Obesity	0.81 (0.25–2.66)	0.733	—	—
Cardiovascular disease	1.09 (0.53–2.28)	0.81	—	—
In‐hospital infection	1.45 (0.56–3.74)	0.443	—	—
Chemotherapy	1.69 (0.87–3.28)	0.122	2.46 (1.15–5.25)	**0.0200**

*Note*: Bold values indicates statistically significant.

Abbreviations: ALC, absolute lymphocyte count; HR, hazard ratio; NLR, neutrophil‐to‐lymphocyte ratio; UNL, upper normal limit.

Other laboratory variables associated with an increased risk of death in the univariable analysis were creatinine and D‐dimer (Table 1 **)**. Patients with creatinine levels > UNL had a lower survival (HR: 2.22; 95% CI: 1.07–4.64). Patients with D‐dimer levels between 1105.30–2411.87 ng/ml (second tertile; HR: 3.59; 95% CI: 1.30–9.89) and D‐dimer levels >2411.87 ng/ml (third tertile; HR: 3.79; 1.38–10.45) have a higher risk of death than patients with D‐dimer levels <1105.30 mg/dl (first tertile). Age ≥ 65 years (HR: 2.33; 95% CI: 1.19–4.59) was also associated with an increased risk of death. Lymphopenia (<1000 cells/mm^3^) did not significantly correlate with clinical outcomes HR 2.18; 95% CI: 0.99–4.80.

After adjusting a multiple Cox regression model including factors with *p*‐values <0.20 as independent variables, NLR > 8.30 persisted associated with an increased risk of death compared to patients with NLR < 3.82 (HR: 3.33; 95% CI: 1.23–8.99) and NLR between 3.82 and 8.30 (HR: 2.77; 95% CI: 1.18–6.53).

Other variables associated with an increased risk of death in the multivariable analysis were D‐dimer and treatment with chemotherapy within 30 days from COVID‐19 diagnosis **(**Table 1 **)**. After multivariable adjustment, patients with D‐dimer levels between 1105.30–2411.87 ng/ml (HR: 4.46; 95% CI: 1.49–13.39) and D‐dimer levels >2411.87 ng/ml (HR: 3.81; 95% CI: 1.25–11.64) had a higher risk of death than patients with D‐dimer levels <1105.30 mg/dl. Treatment with chemotherapy within 30 days from COVID‐19 diagnosis (HR: 2.46 (1.15–5.25)) was associated with an increased risk of death.

## 4 DISCUSSION

^.^ In this study, patients with solid tumors with higher NLR values had worse clinical outcomes. NLR is an inexpensive and almost always available prognostic biomarker in COVID‐19, related to the combination of lymphopenia and neutrophilia associated with progression to critical disease.^8^ Neutrophilia, although a defensive response by the immune system, if not optimally regulated, can lead to concurrent tissue damage.^26^ Neutrophils utilize multiple defense mechanisms including phagocytosis, degranulation, cytokine production, and newly described ability to form neutrophil extracellular traps (NETs).^22^ NETs, are web like structures, comprising of DNA and proteins, which are released from neutrophils to entrap pathogens.^27^ Unrestricted NET formation can lead to an inflammatory cascade resulting in an irreversible tissue injury to the pulmonary, cardiovascular and renal systems^28^, ^29^, ^30^ explaining the increased mortality in COVID‐19 patients.^31^, ^32^ NETs have also been associated with acute respiratory distress syndrome (ARDS) and cytokine storm, both well‐known mechanisms contributing to mortality in COVID‐19 patients.^23^, ^33^

Similar to our study, NLR has been reported as a poor prognostic factor in patients with COVID‐19.^8,34^ However, majority of the studies related to prognostic factors in COVID‐19 patients included non‐oncological patients. In a UK cohort of 302 cancer patients, including 86 cases of hematological cancer, higher NLR at day 0 was a predictor of death.^35^ In another study by Calles et al., consisting of only lung cancer patients, the median of the NLR at diagnosis was higher in high‐risk patients.^36^

In our study, we found that NLR > 8.30 was associated with worse outcomes in solid tumor patients. Various studies have tried to define optimal cutoff values for the NLR ratio. In a meta‐analysis consisting of 38 studies, elevated NLR was positively associated with severity and mortality. However, they did not find a cutoff value to define prognosis.^37^ Studies that used the NLR as a qualitative variable, different cutoff values were utilized. A NLR equal or higher than 2.97 was an independent factor for progression to severe or critical disease in 301 cases in China.^38^ Cai et al. found a NLR of 6.11 as a cutoff value in the receiver operating characteristic (ROC) curve to discriminate prognosis. The area under the ROC curve of the NLR was higher than each of the neutrophils or lymphocyte curves separately.^6^ Calles et al. specifically in the lung cancer patients, found the optimal cutoff value for differentiating low‐ versus high‐risk patients was 10.69.^36^ Prospective and larger studies are needed to define an optimal cut off, which might vary with existing comorbidities like cancer.

There were several limitations of our study. First, it was a single center, retrospective study which included patients from a cancer referral hospital. Second, our study included patients of the first two waves of the pandemic in Argentina and the different strains of SARS‐Cov‐2 might have different impact over morbidity or mortality. Another important consideration is the vaccination rate. In Argentina the vaccination campaign started at the end of February 2021 and most patients in this study were unvaccinated. Due the retrospective nature of the study we were unable to capture the treatment received and complications related to COVID‐19, which could also potentially impact mortality in patients.

From clinical practical perspective, it would also be interesting to monitor NLR in therapeutics targeting specifically neutrophils and its components. Several studies targeting NETs or their triggers, namely IL17,^39^ gasdermin D,^40^ peptidyl arginine deiminase type 4^41^ and neutrophil elastase^42^ are now in experimental phases for COVID‐19. NLR could also be a useful adjunct in such clinical trials.

In our study, a higher NLR value at hospital admission was associated with mortality in solid cancer patients. It is an inexpensive and readily available biomarker. Prospective studies, with a larger cohort are needed to further validate and define specific cutoff values for the NLR in cancer patients affected with COVID‐19.

## AUTHOR CONTRIBUTIONS

**Fernando A. Díaz‐Couselo:** Conceptualization (equal); data curation (equal); formal analysis (equal); methodology (equal); validation (equal); writing – original draft (equal); writing – review and editing (equal). **Santiago Flagel:** Data curation (equal); formal analysis (equal). **Carla Nicolini:** Data curation (equal). **Sebastián Halac:** Data curation (equal). **Natalia Manzano:** Data curation (equal). **Marina Aguirre:** Data curation (equal). **Juan Rébora:** Data curation (equal). **Sandra Valle:** Formal analysis (equal); investigation (equal); methodology (equal). **Laura Noro:** Formal analysis (equal); investigation (equal); methodology (equal). **Chirayu Mohindroo:** Methodology (equal); writing – original draft (equal); writing – review and editing (equal). **Florencia McAllister:** Formal analysis (equal); investigation (equal); supervision (equal); visualization (equal); writing – original draft (equal); writing – review and editing (equal). **Marcelo O. Zylberman:** Conceptualization (equal); formal analysis (equal); investigation (equal); methodology (equal); supervision (equal); visualization (equal); writing – original draft (equal); writing – review and editing (equal).

## CONFLICT OF INTEREST

FM is an SAB member at Neologics Bio.

## Supporting information


Table S1

Table S2

Appendix S1

## Data Availability

Data availability statement: The data that support the findings of this study are available on request from the corresponding author.
