# Supplementary material for: Impact of high neutrophil‐to‐lymphocyte ratio on survival in hospitalized cancer patients with COVID‐19
Source: Cancer Med. 2022 Nov 13;12(6):7164–9. doi: 10.1002/cam4.5426 (PMC9877941; doi:10.1002/cam4.5426)
Supplement: Supplementary file 1 — Table S1 [file CAM4-12-7164-s001.docx]

**Supplementary Table 1.** Cancer types included in the study.

| **Tumor Type** | N (%) |
| --- | --- |
| Breast | 25 (20.8) |
| Lung | 24 (20.0) |
| Colorectal | 19 (15.8) |
| Head and Neck | 6 (5.0) |
| Ovarium | 6 (5.0) |
| Bladder | 5 (4.2) |
| Cervix | 4 (3.3) |
| Melanoma | 4 (3.3) |
| Pancreas | 3 (2.5) |
| Prostate | 3 (2.5) |
| Anal | 2 (1.7) |
| Cholangiocarcinoma | 2 (1.7) |
| Esophagus | 2 (1.7) |
| Gastric | 2 (1.7) |
| Germ cell | 2 (1.7) |
| Renal | 2 (1.7) |
| Soft tissue sarcoma | 2 (1.7) |
| Endometrium | 1 (0.8) |
| Small intestine | 1 (0.8) |
| Unknown origin | 1 (0.8) |
| Lung and renal | 1 (0.8) |
| Central nervous system | 1 (0.8) |
| Thymoma | 1 (0.8) |
| Gallbladder | 1 (0.8) |
