# Supplementary material for: Impact of high neutrophil‐to‐lymphocyte ratio on survival in hospitalized cancer patients with COVID‐19
Source: Cancer Med. 2022 Nov 13;12(6):7164–9. doi: 10.1002/cam4.5426 (PMC9877941; doi:10.1002/cam4.5426)
Supplement: Supplementary file 2 — Table S2 [file CAM4-12-7164-s003.docx]

**Supplementary Table 2.** Clinical information of patients included in the the study.

| **Variable** |  |  |  |
| --- | --- | --- | --- |
| Age in years, median (IQR) | 62.5 (54–71) |  |  |
| ≥ 65 years, n (%) | 51 (42.5%) |  |  |
| Male, n (%) | 69 (57.5%) |  |  |
| Stage |  |  |  |
| No distant metastasis | 27 (22.5%) |  |  |
| Locally advanced | 17 (14.2%) |  |  |
| Metastatic disease | 76 (63.3%) |  |  |
| Smoking history, n (%) | 40 (33.3%) |  |  |
| Hypertension, n (%) | 45 (37.5%) |  |  |
| Hypercholesterolemia, n (%) | 24 (20.0%) |  |  |
| Obesity, n (%) | 13 (10.8%) |  |  |
| Diabetes, n (%) | 19 (15.8%) |  |  |
| Cardiovascular disease | 33 (27.5%) |  |  |
| Chemotherapy | 45 (37.5%) |  |  |
| In-hospital infection, n (%) | 14 (11.7%) |  |  |
| ICU requirement | 46 (38.3%) |  |  |
| Leukocytes/mm^3^, median (IQR) | 6,200 (4,375–9,825) |  | Ref: 4,800–10,800 |
| Neutrophils/mm^3^, median (IQR) | 4,449 (2,882–7,960) |  |  |
| Lymphocytes/mm^3^, median (IQR) | 788 (517–1,149) |  |  |
| ALC < 1000/mm^3^, n (%) | 76 (63.3%) |  |  |
| NLR, median (IQR) | 5.06 (2.83–10.25) |  |  |
| Creatinine in mg/dl, median (IQR) | 0.79 (0.67–1.01) |  | Ref: 0.57–1.11 (female); 0.72-1.25 (male) |
| LDH in U/l, median (IQR) | 295.5 (204.8–382.8) |  | Ref: ≤220 |
| D-dimer in ng/ml, median (IQR) | 1,736.6 (959.4–3,178.7) |  | Ref: ≤ 500 |
| Ferritin in ng/ml, median (IQR) | 931.9 (455.7–1,775.7) |  | Ref: 4.63 – 204 |
| CRP in mg/l, median (IQR) | 74.6 (28.3–142.9) |  | Ref: ≤ 5 |
