# Supplementary material for: Impact of high neutrophil‐to‐lymphocyte ratio on survival in hospitalized cancer patients with COVID‐19
Source: Cancer Med. 2022 Nov 13;12(6):7164–9. doi: 10.1002/cam4.5426 (PMC9877941; doi:10.1002/cam4.5426)
Supplement: Supplementary file 3 — Appendix S1 [file CAM4-12-7164-s002.docx]

**Supporting information**

**Statistical analysis**

Missing data analysis was performed. Variables were discarded if there were more than 20% of missing values. Missing data imputation was done by multiple imputation of chained equations [1]. The results of the categorical variables are reported as frequencies and percentages. The results of the quantitative variables are expressed as median and interquartile range (IQR). Survival plots were obtained estimating the probability of survival by the method of Kaplan-Meier. For quantitative variables, univariable Cox models were tested for the assumption of linearity between the log hazard and each covariate by residual plots.

As linearity was not verified, variables were categorized. Age was categorized with a cutoff value of 65 years, as medical literature used values between 60 and 70 years [2-5] Serum creatinine was dichotomized according to its upper normal limit [6] (UNL; female: 1.11 mg/dl; male: 1.25 mg/dl; Abbott Laboratories, IL, USA; Alinity c, Creatinine Reagent Kit; 2018). Lymphopenia was defined as an absolute lymphocyte count < 1000 cells/mm^3^ [7]. Other quantitative variables, as there is no consensus of cut-off values, particularly in cancer patients, were categorized in tertiles. Proportionality was verified with deviance residuals analysis.

A multivariable Cox model was adjusted with independent variables with p-values < 0.2 in the univariable analysis. Statistical analysis was performed with R (R Core Team) using the Integrated Development Environment R Studio.

**References**

1. Van Buuren, S. and K. Groothuis-Oudshoorn, *mice: Multivariate imputation by chained equations in R.* Journal of statistical software, 2011. **45**: p. 1-67.

2. Albiges, L., et al., *Determinants of the outcomes of patients with cancer infected with SARS-CoV-2: results from the Gustave Roussy cohort.* Nature Cancer, 2020. **1**(10): p. 965-975.

3. Chen, L., et al., *Risk factors for death in 1859 subjects with COVID-19.* Leukemia, 2020. **34**(8): p. 2173-2183.

4. Garassino, M.C., et al., *COVID-19 in patients with thoracic malignancies (TERAVOLT): first results of an international, registry-based, cohort study.* The Lancet Oncology, 2020. **21**(7): p. 914-922.

5. Jee, J., et al., *Chemotherapy and COVID-19 outcomes in patients with cancer.* Journal of Clinical Oncology, 2020. **38**(30): p. 3538-3546.

6. AO, H., *Walker HK, Hall WD, Hurst JW, editors. BUN and creatinine.* Clinical methods: the history, physical, and laboratory examinations. 3rd ed. Boston: Butterworths, 1990.

7. Lee, J., et al., *Lymphopenia as a biological predictor of outcomes in COVID-19 patients: a nationwide cohort study.* Cancers, 2021. **13**(3): p. 471.
